# Immune mediated therapy and MEK inhibition: preclinical assessment of immunobiology and combination activity in vitro and in vivo

**DOI:** 10.1186/2051-1426-2-S3-P128

**Published:** 2014-11-06

**Authors:** Ross Stewart, Edmund Poon, Stefanie Mullins, Amanda Watkins, Paul D Smith, Robert W Wilkinson

**Affiliations:** 1 MedImmune, Cambridge, UK; 2 Astra Zeneca, Macclesfield, UK

## 

Immune mediated therapies (IMT), such as anti-CTLA-4 and anti-PD-1/PD-L1, are showing significant promise in the treatment of solid tumors. However, although these treatments can show significant overall benefit, a subset of patients fails to respond. It is believed that activity in these patients is limited by a lack of immune priming or by immunosuppression. Combination with molecular targeted therapies has the potential to overcome these hurdles to response and maximize patient benefit. In order to select the best combination partners, a greater understanding is needed of how other therapies affect the immune system both directly, through effects on leukocytes, and indirectly, through effects on tumor immunogenicity and induction of tumor cell death.

Selumetinib (AZD6244, ARRY-142886) is a potent inhibitor of the mitogen-activated protein/extracellular signal-regulated kinase kinases 1/2 (MEK1/2), involved in the activation of signaling pathways which regulates cellular growth, proliferation and survival. Given that these pathways are often found aberrantly activated in human tumours and also play key roles in the regulation of immunological processes, it is difficult to predict the combinatorial effect of MEK1/2 inhibition and IMT. Here we detail a systematic approach taken towards examining the effects of MEK1/2 inhibition on tumour immunogenicity *in vitro *and *in vivo *and the direct effects it may have on the function of the immune system. We also present preclinical data demonstrating the anti-tumour activity of combining selumetinib and IMT mAbs in a syngeneic mouse model and report on possible mechanisms of synergism in promoting an anti-tumour immune response through promotion of antigen presentation.

